# Blood Transfusion Risk Following Early Versus Delayed Surgery in Hip Fracture Patients on Direct Oral Anticoagulants: A Study Protocol for a Natural Experiment

**DOI:** 10.3390/jcm15020758

**Published:** 2026-01-16

**Authors:** Tim Schiepers, Diederik Smeeing, Hugo Wijnen, Hanna Willems, Frans Jasper Wijdicks, Elvira Flikweert, Diederik Kempen, Eelke Bosma, Johannes H. Hegeman, Marielle Emmelot-Vonk, Detlef van der Velde, Henk Jan Schuijt

**Affiliations:** 1 Department of Trauma Surgery, St. Antonius Hospital, 3543 AZ Utrecht, The Netherlands; 2 Department of Geriatrics, University Medical Center Utrecht, Utrecht University, 3584 CX Utrecht, The Netherlands; 3 Department of Trauma Surgery, Rijnstate Hospital, 6800 TA Arnhem, The Netherlands; 4 Department of Geriatrics, Rijnstate Hospital, 6800 TA Arnhem, The Netherlands; 5 Internal Medicine and Geriatrics, Amsterdam UMC Location, University of Amsterdam, 1105 AZ Amsterdam, The Netherlands; 6 Department of Trauma Surgery, Diakonessenhuis Hospital, 3508 TG Utrecht, The Netherlands; 7 Department of Trauma Surgery, Deventer Hospital, 7416 SE Deventer, The Netherlands; 8 Department of Orthopedic Surgery, OLVG Hospital, 1091 AC Amsterdam, The Netherlands; 9 Department of Trauma Surgery, Martini Hospital, 9728 NT Groningen, The Netherlands; 10 Department of Trauma Surgery, ZGT Hospital, 7609 PP Almelo, The Netherlands

**Keywords:** hip fracture, anticoagulants, blood transfusion, natural experiment, time to treatment, treatment outcome, postoperative complications

## Abstract

**Background**: Early surgical intervention is associated with improved outcomes in hip fracture care, yet in patients using Direct Oral Anticoagulants (DOACs), surgery is frequently delayed due to concerns about increased intraoperative bleeding. Despite the increasing prevalence of hip fracture patients on DOACs, no consensus exists on optimal surgical timing. This has led to substantial practice variation between hospitals, with some operating within 24 h of last DOAC intake and others delaying surgery beyond 24 h. This study hypothesizes that early surgery within 24 h results in a non-inferior blood transfusion risk compared to delayed surgery 24 h or more after last DOAC intake in hip fracture patients on DOACs. This protocol describes the design and methodological rationale of a natural experiment. **Methods and analysis**: A multicenter cohort study designed as a natural experiment will be conducted across seven Dutch level 2 trauma centers, using predefined and standardized prospectively collected variables from electronic health records. Centers will adhere to distinct local surgical timing protocols, forming two cohorts: early surgery within 24 h and delayed surgery 24 h or more after last DOAC intake. Patients presenting with an isolated hip fracture who are using a DOAC and have taken their last dose within 24 h before admission will be included. The primary endpoint is postoperative blood transfusion. Secondary endpoints include additional bleeding-related outcomes, thrombotic and postoperative complications, and hospital length of stay. The primary analysis will be conducted on a per-protocol basis, with an intention-to-treat analysis performed as a supplementary assessment. Non-inferiority will be established if the upper bound of the one-sided 95% confidence interval for the risk difference does not exceed the predefined margin of 5%. **Ethics and dissemination**: Ethical approval was obtained from the Medical Ethics Committee United, Utrecht, The Netherlands. As this is a cohort study without altering clinical care, individual informed consent is not required. All data will be pseudonymized, and findings will be disseminated through peer-reviewed journals and scientific conferences. **Registration details**: Medical Ethics Committee United, Utrecht, The Netherlands, registration number W25.034.

## 1. Strengths and Limitations

- This study addresses an urgent and clinically relevant knowledge gap regarding the optimal surgical timing for hip fracture patients on direct oral anticoagulants, with the potential to improve national and international guidelines.
- It utilizes a natural experiment design across seven Dutch trauma centers, leveraging existing practice variation to approximate randomization and enable real-world comparison of early surgery within 24 h versus delayed surgery 24 h or more after last direct oral anticoagulant intake, without interfering with routine clinical care, enhancing the generalizability of the findings.
- While the retrospective data extraction from electronic health records at predefined intervals is a limitation, all variables are prospectively predefined and standardized across participating hospitals, and regular data quality checks with structured feedback to centers are implemented to ensure consistency, completeness, and high data quality.
- Due to the limited number of covariates allowed in the regression model to prevent overfitting, there remains a risk of residual confounding; however, the most clinically relevant confounders will be accounted for in the analysis.
- A per-protocol primary analysis may be susceptible to confounding by indication; however, a pre-planned intention-to-treat analysis will assess the robustness of findings and mitigate this bias. In addition, time-dependent confounding cannot be fully excluded. Clinical or logistical factors arising during the preoperative waiting period, such as medical optimization, complications, or operating room availability, may influence both surgical timing and outcomes independent of direct oral anticoagulant status.

## 2. Introduction

Early surgical intervention is the standard of care for hip fractures, and there is robust evidence that shorter time to surgery improves patient outcomes, whereas delays are associated with higher mortality and worse outcomes, including prolonged immobility, extended hospital length of stay (HLOS), and greater patient discomfort [1,2,3,4,5,6,7,8]. However, in hip fracture patients on direct oral anticoagulants (DOACs), surgery is often postponed due to concerns about increased intraoperative bleeding risk while the anticoagulant is still active [9,10,11]. Although reversal agents for DOACs such as idarucizumab and andexanet alfa are now available, their high cost has limited their widespread adoption [12,13]. 

The clinical importance of this dilemma is particularly relevant given that hip fractures are among the most common causes of hospital admission in older patients and are associated with high mortality and a loss of independence [14]. Nationwide registry data indicate that nearly 20,000 hip fractures occur annually in The Netherlands [15]. Hip fracture patients frequently have multiple comorbidities and cognitive disorders, further increasing their vulnerability to poor outcomes [16,17,18]. Cardiovascular disease requiring long-term anticoagulation is particularly common, and DOACs have largely replaced vitamin K antagonists and heparins due to their predictable pharmacokinetics, fixed dosing, and limited monitoring requirements [19,20,21,22,23,24,25,26]. Currently, 12–16% of hip fracture patients require long-term anticoagulation with DOACs [19,20,21]. DOACs target different sites in the coagulation cascade, either as direct factor Xa inhibitors (apixaban, edoxaban, rivaroxaban) or as a direct factor IIa inhibitor (dabigatran) [27]. Their half-life is typically 10–14 h but may be prolonged up to 27 h in older patients due to reduced renal clearance and altered distribution, adding further uncertainty on surgical timing decisions (Appendix A; Table A1) [27,28]. This is particularly relevant for dabigatran, which is predominantly cleared renally [27,28].

Despite its clinical relevance, no consensus exists in the literature on the optimal surgical timing after DOAC discontinuation in hip fracture patients [12,27,29]. The uncertainty about this dilemma is reflected in clinical practice: 74% of surgeons consider current guidelines for hip fracture patients on DOACs inadequate, contributing to substantial clinical practice variation across hospitals [12,13,30,31]. Consequently, some hospitals postpone surgery for at least 48 h to mitigate bleeding risk, and some determine the timing of surgery based on renal function [12,13,30]. Retrospective studies suggest that early surgery may be safe; however, only one single-center study has directly compared the outcome of surgery performed within 24 h to after 24 h or more [27,32,33,34]. Nonetheless, expert consensus in several Dutch hospitals now permits immediate surgery for hip fracture patients on DOACs, as concerns also extend to preoperative blood loss from ongoing bleeding in unstabilized fractures [35,36,37].

This study will evaluate whether surgery within 24 h after last DOAC intake is as safe as surgery after 24 h or more in hip fracture patients on DOACs with respect to bleeding risk. Postoperative blood transfusion was chosen as the primary outcome because it represents a clinically relevant and objectively recorded outcome in routine practice. The hypothesis is that early surgery within 24 h results in a non-inferior postoperative blood transfusion risk compared to delayed surgery 24 h or more after last DOAC intake in hip fracture patients on DOACs. This protocol article outlines the design and methodological rationale of a natural experiment.

## 3. Clinical Relevance

The findings of this study will be particularly significant for the hip fracture population for several reasons. There is currently no consensus on the optimal timing of surgery for hip fracture patients on DOACs, leading to considerable clinical practice variation in discontinuation protocols across The Netherlands [12,13,27,30,31]. Clear, evidence-based recommendations are urgently needed to standardize care and improve outcomes in this vulnerable and growing patient population [31,38].

If early surgery is shown to be non-inferior with respect to bleeding and thrombotic complications, this would support a paradigm shift toward performing hip fracture surgery within 24 h of last DOAC intake. This could lead to reduced hospital length of stay, faster mobilization and rehabilitation, and lower the risk of complications related to immobilization such as pressure ulcers and nosocomial infections [39,40]. Moreover, the reduction in HLOS could lead to significant cost savings for healthcare systems, as well as improved capacity and resource utilization [40,41].

If early surgery is proven inferior to delayed surgery, this study will help to define the optimal timing of surgery after DOAC discontinuation, possibly based on renal function and drug clearance. This could enable widespread (inter)national implementation of standardized perioperative protocols, ultimately contributing to evidence based, safe, and more efficient hip fracture care.

## 4. Objectives

### 4.1. Primary Objective

To determine whether early surgery within 24 h after last DOAC intake compared with delayed surgery after 24 h or more results in a non-inferior blood transfusion risk in hip fracture patients on DOACs, where non-inferiority is defined as the upper bound of the one-sided 95% confidence interval for the absolute risk difference remaining below a predefined margin of 5%.

### 4.2. Secondary Objectives

- To investigate the effect on other bleeding-related outcomes in this population (i.e., preoperative blood transfusions, pre- and postoperative hemoglobin difference, the bleeding index, amount of packed red blood cells administered, reoperation due to postoperative bleeding or infection, sciatic neuropraxia due to postoperative bleeding or infection, postoperative anemia and postoperative hematoma).
- To investigate the effect on thromboembolic outcomes in this population (such as acute myocardial infarction, stroke, pulmonary embolism, peripheral arterial thrombosis, and deep venous thrombosis).
- To investigate the effect on postoperative complications in this population (i.e., wound fluid leakage, pressure ulcer, congestive heart failure, delirium, renal insufficiency, pneumonia, urinary tract infections, deep or superficial surgical wound infection, and in-hospital falls).
- To investigate the effect on in-hospital and 30-day mortality in this population.
- To investigate the effect on hospital length of stay and discharge destination in this population.

## 5. Materials and Methods

### 5.1. Study Design

This cohort study is designed as a natural experiment and includes seven level 2 trauma centers across The Netherlands. Participating hospitals follow either an expert-based protocol permitting surgery within 24 h after the last DOAC intake or a conservative protocol requiring a minimum delay of 24 h. Patient inclusion starts in November 2025 and is expected to conclude in June 2027.

### 5.2. Natural Experiment

#### 5.2.1. Rationale

Natural experiments are observational studies in which treatment allocation is determined by external factors rather than by investigators, closely resembling the principles of randomization [42]. In the context of orthopedic trauma, natural experiments remain relatively uncommon, but offer significant potential due to existing variability in treatment strategies between hospitals or regions [42]. These differences often stem from local education, institutional culture, and expert opinion [42]. By leveraging these naturally occurring variations, this study aims to evaluate treatment outcomes without altering clinical practice.

This study follows the principles outlined by the Natural Experiments (NEXT) Study Group, which provides methodological guidance for designing natural experiments in trauma research [42]. Key design features include the use of distinct “schools of treatment” where institutional protocols consistently determine care, while patient allocation to hospitals occurs independently of individual characteristics [42]. To support comparability between groups, all participating hospitals are level 2 trauma centers with similar hip fracture volumes, staffing, and standardized perioperative care. These similarities reduce the chance that outcome differences are due to institutional differences.

Importantly, this study constitutes a Type 2 Comparison in orthopedic trauma surgery, as defined by Beks et al., in which two active treatment strategies (early versus delayed surgery) are compared for the same clinical indication [43]. Observational studies are particularly well suited for this type of comparison when treatment allocation is determined by institutional protocols or surgeon preference rather than individual patient characteristics [43,44]. This scenario is common in trauma care, where patients are often assigned to hospitals or surgical teams based on logistical factors, resulting in quasi-randomization in treatment [42,43]. This quasi-randomization reduces the effect of confounding and selection bias and strengthens the validity of the comparisons [42,43]. Nevertheless, as with all observational designs, the possibility of residual unmeasured confounding cannot be entirely excluded [43,44,45].

Despite this, well-designed observational studies, particularly natural experiments, can yield results similar in credibility to randomized controlled trials (RCTs) [43,44]. In addition, RCTs are often impractical or unethical in trauma research due to the urgency of treatment decisions, challenges in obtaining informed consent in acutely injured or cognitively impaired patients, and the logistical difficulties of randomization in acute care settings [42,43]. Natural experiments thus represent a methodologically sound and ethically viable alternative for evaluating treatment effectiveness in real-world trauma care [42,43].

#### 5.2.2. Treatment Allocation

**Figure A1:**
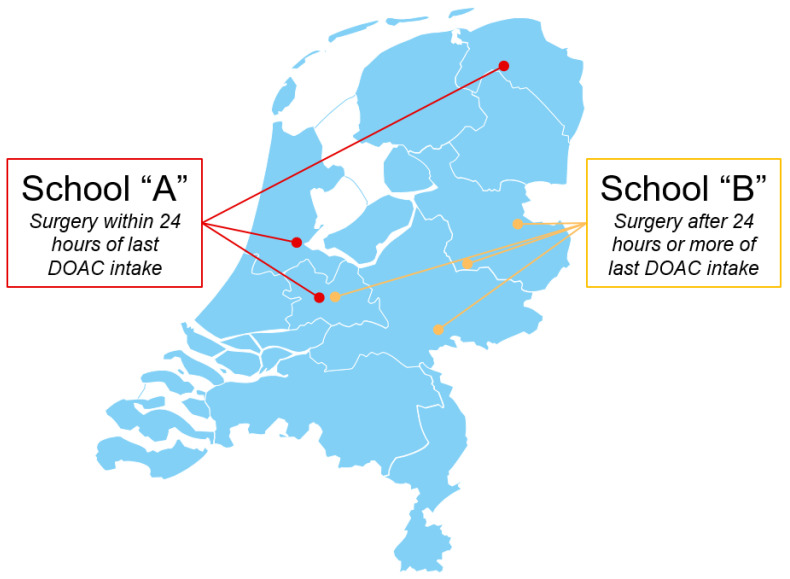
Geographical overview of participating hospitals in The Netherlands.

Participating hospitals are divided into two cohorts based on the pre-existing “schools of treatment”, reflecting distinct institutional protocols and inherent differences in the clinical management of hip fracture patients on DOACs [42]. School “A” (early surgery cohort) comprises three hospitals and School “B” (delayed surgery cohort) four hospitals, ensuring broad geographic distribution across The Netherlands (Appendix A; Figure A1). In the hospitals belonging to School “A”, the standing policy is to perform surgery on hip fracture patients on DOACs within 24 h after last DOAC intake. In contrast, hospitals in School “B” follow a strict protocol requiring a delay of at least 24 h after last DOAC intake before surgery to allow for DOAC clearance. One participating hospital applies a mixed protocol: delaying prosthesis surgery for 24 h after the last DOAC intake while performing all other procedures within 24 h. This setting still constitutes a natural experiment because allocation to early or delayed surgery is determined solely by the local institutional protocol rather than by surgeon, patient, or researcher intervention. In rare cases where deviation from the protocol occurs, for example, due to medical urgency in School “B” or logistical or clinical reasons in School “A”, these patients will be identified and handled accordingly in the analyses. The analytic plan for handling these deviations is further described in Section 5.8.

#### 5.2.3. Policy Changes

Initially, allocation to these groups occurs naturally based on the hospital where the patient is admitted, rather than by investigator intervention, ensuring that treatment allocation is independent of individual patient characteristics. However, in the unlikely event that during the study (orthopedic) trauma surgeons within School “B” change their protocol and start performing surgery within 24 h after last DOAC intake, these patients will be included in School “A”. Conversely, if surgeons within School “A” change their protocol to delay surgery at least 24 h after last DOAC intake, patients from that point onward will be included in School “B”. In both cases, allocation will then be based on surgeon-level protocol rather than hospital-level standard practice. Despite this, the study remains a natural experiment, as patients continue to be quasi-randomly allocated to comparable level 2 trauma centers with uniform hospital resources and patient populations.

### 5.3. Eligibility Criteria

**Figure 1:**
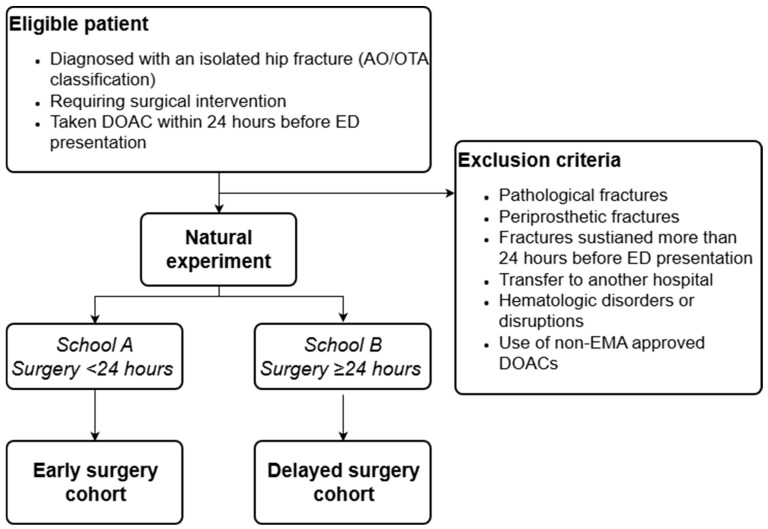
Flowchart of the inclusion process in a natural experiment design. This flowchart outlines the structure of the natural experiment comparing early surgery within 24 h versus delayed surgery 24 h or more after last DOAC intake in hip fracture patients on DOACs. Eligible patients are quasi-randomly allocated based on hospital treatment protocol (School “A” or School “B”), with inclusion and exclusion criteria displayed.

Patients are eligible for inclusion if they present with an isolated hip fracture classified as AO/OTA 31A or 31B, requiring surgical intervention, are actively using DOACs and have taken their DOAC within 24 h before ED presentation (Figure 1) [46]. Exclusion criteria include pathological or periprosthetic fractures, fractures sustained more than 24 h before ED presentation, and transfers to another hospital. Patients with hematologic disorders or disruptions (i.e., thalassemia, sickle cell disease, aplastic anemia, myelodysplastic syndromes, or leukemia) will also be excluded, as will those using a non-approved DOAC (i.e., betrixaban) by the European Medicines Agency. Patients presenting to the ED more than 24 h after fracture occurrence are excluded, as delayed presentation introduces uncertainty regarding active DOAC exposure in the early surgery cohort. In such cases, anticoagulant activity may have substantially declined irrespective of institutional surgical timing protocols. Although this exclusion may limit generalizability and introduce selection bias, it was considered necessary to preserve internal validity and ensure meaningful comparability between early and delayed surgery cohorts.

### 5.4. Baseline Characteristics

The following baseline characteristics will be collected: age, sex, preoperative estimated glomerular filtration rate (eGFR in mL/min/1.73 m^2^), preoperative hemoglobin (Hb in mmol/L), preoperative hematocrit (Ht in L/L), type of DOAC (apixaban, edoxaban, rivaroxaban, or dabigatran), DOAC dosage, DOAC indication, hours since last DOAC intake, concurrent use of antiplatelet medication (yes/no), and the specific type of concurrent antiplatelet or anticoagulant medication used. Frailty indicators include a prior diagnosis of dementia (by a geriatrician or general practitioner), Body Mass Index (BMI in kg/m^2^), Short Nutritional Assessment Questionnaire (SNAQ) score, Clinical Frailty Scale (CFS), Charlson Comorbidity Index (CCI), American Society of Anesthesiologists (ASA) score, number of daily medications, pre-fracture living situation (independently at home, with assistance in Activities of Daily Living (ADL), or in institutional care facility), household composition (living alone, with a partner, or with family), pre-fracture mobility (based on the Fracture Mobility Score), and Katz-6 ADL score [47,48,49,50,51,52]. Fracture characteristics include the type of fracture (based on AO/OTA classification) and the surgical procedure performed (hemiarthroplasty, total hip arthroplasty, intramedullary nail, sliding hip screw, or cannulated hip screw) [46]. Lastly, the type of anesthesia (general or spinal), any preoperative use of tranexamic acid, fibrinogen, or platelet concentrates, and DOAC reversal agents (idarucizumab or andexanet alfa) will be registered. In addition, patients whose surgery was delayed for reasons unrelated to DOAC use will be documented, along with the specific reason for the delay. Measurement of DOAC plasma levels is not part of routine care in the participating centers and is therefore not included.

### 5.5. Outcomes

#### 5.5.1. Primary Outcome

The primary outcome of this study is postoperative blood transfusion, defined as the administration of one or more units of packed red blood cells (PRBCs) postoperatively during admission. This outcome serves as a robust, objective, and clinically relevant marker of perioperative blood loss. Transfusion is a widely used endpoint in studies evaluating surgical bleeding, as it reflects a clinical decision based on pre-, intra- and postoperative hemodynamics, Hb thresholds, and clinical symptoms. Unlike subjective measures such as hematoma grading or intraoperative blood loss estimates, transfusion requirement provides a clear, binary outcome with direct clinical implications for patient recovery and safety. Inter-hospital variation in transfusion practices is likely to be minimal, because all participating hospitals adhere to the 4–5–6 rule or the Dutch national transfusion guideline (2020) [53]. Although minor differences in transfusion policies may exist between participating centers, these are expected to be evenly distributed across study cohorts and these will not be altered to preserve the natural experiment design. Moreover, variability at the level of individual providers is inherent to routine clinical care and is expected to occur similarly across all participating centers, making systematic differences between groups unlikely. In conclusion, any inter-hospital variation in transfusion thresholds is expected to be non-differential between the early and delayed surgery cohorts, as transfusion decisions are not systematically related to surgical timing. Such non-differential variability would be expected to bias effect estimates toward the null, thereby reducing the risk of falsely concluding non-inferiority.

#### 5.5.2. Secondary Outcomes

Secondary outcomes include a broad range of bleeding-related outcomes, thrombotic complications, and postoperative complications, and healthcare resource utilization. Bleeding-related outcomes include preoperative blood transfusion, the number of PRBCs administered (1, 2, 3, or ≥4 units), lowest postoperative Hb and Ht levels within 24 h postoperatively, Hb decrease (ΔHb) (defined as the difference between preoperative Hb (measured at the ED) and the lowest postoperative Hb within 24 h), the bleeding index (defined as ΔHb plus the number of PRBC transfused between the ΔHb period), the number of patients experiencing a Hb decrease of more than 2 mmol/L. Estimated intraoperative blood loss will not be recorded, as it is considered too subjective, prone to outcome reporting bias, and highly susceptible to inter-rater variability. Clinical complications such as reoperation due to postoperative bleeding or infection, sciatic neuropraxia due to postoperative bleeding or infection, postoperative hematoma, and postoperative anemia will also be recorded. Lastly, administration of tranexamic acid, fibrinogen, or platelet concentrates will also be assessed.

Thrombotic complications recorded in this study comprise myocardial infarction (confirmed by electrocardiogram), stroke (confirmed by CT), pulmonary embolism (confirmed by CT-Angiography), deep vein thrombosis (confirmed by ultrasound), and peripheral arterial thrombosis (confirmed by CT-Angiography).

Other postoperative complications as determined by the Dutch Hip Fracture Audit guidelines will be assessed and include pressure ulcers, delirium, deep or superficial surgical wound infections, wound fluid leakage, days of wound fluid leakage, congestive heart failure, and in-hospital falls (all diagnosed by the attending physician or geriatrician) [54]. Additional complications include pneumonia (confirmed by chest radiograph or positive sputum culture), urinary tract infections (confirmed by a positive urine culture), renal insufficiency (defined as a >24 mL/min/1.73 m^2^ decrease in eGFR compared to admission), in-hospital mortality (recorded in hospital records), and 30-day mortality (determined through municipality records) [54].

Additional secondary outcomes include: hospital length of stay (in days, defined as the time between ED presentation and discharge from hospital or in-hospital death), time to surgery (in hours, from ED presentation to start of surgery), hours between last DOAC intake and start of surgery (in hours, defined as the interval between the last reported DOAC intake and the start of surgery), time until DOAC therapy is restarted after surgery (in hours, defined as the time between end of surgery and first postoperative DOAC dose), total time without DOAC therapy (in hours, defined as the time between last DOAC intake and the first postoperative DOAC dose), surgical duration (in minutes, recorded from incision to closure), and discharge destination (categorized as: home, home with assistance for ADL, temporary skilled nursing care, geriatric rehabilitation care, or a long term geriatric care facility).

### 5.6. Data Management

In this study, data registration will be performed prospectively in the electronic health records (EHRs) of all participating centers, according to a predefined and standardized set of variables. This prospective registration strategy is intended to minimize missing data and ensure uniformity across centers. At several predefined time points during the study period, data will be retrospectively extracted from the EHRs by the executive researcher, who will receive a guest appointment in each of the seven participating centers. If interim quality checks indicate incomplete or inconsistent registration in certain centers, feedback will be provided to optimize data quality during the ongoing study. Thus, while data registration is prospective, data collection for analysis will take place multiple times retrospectively during the inclusion period.

All data will be collected using a standardized protocol and entered into REDCap, in accordance with the FAIR principles (Findable, Accessible, Interoperable, and Reusable) [55]. All data will be securely and confidentially stored on the research network drive of the trauma division at the coordinating study center. Directly identifiable data (e.g., patient ID) will be stored separately in a password-protected Excel file, while research data will be pseudonymized and stored in REDCap, and exported to RStudio (version 2024.04.1, Posit, PBC) for statistical analysis only [56]. A coded key file will enable linkage between the datasets and will be securely stored separately. Only the principal investigator and executive researcher will have access to these files.

Data management procedures will adhere to the European Union General Data Protection Regulation (GDPR) and the Dutch Act on Implementation of the GDPR [57,58]. All data will be retained for 15 years following the end of the study, in line with ethical and legal requirements. The coordinating study center serves as the data controller and is responsible for safeguarding data integrity and security throughout the study period.

### 5.7. Sample Size and Rationale for Non-Inferiority

The research question of this study is framed as a non-inferiority comparison, and the sample size has therefore been calculated to test non-inferiority. This study used the most recent institutional data, as these best reflect the clinical context and patient population of this study. These institutional data showed a transfusion rate of approximately 15% in the delayed surgery group and 10% in the early surgery group, which is considered appropriate for powering a non-inferiority analysis in this natural experiment. In consultation with clinical experts, a non-inferiority margin (ΔNI) of 5% is deemed clinically acceptable to conclude that early surgery does not result in a clinically relevant increase in transfusion requirement. Non-inferiority will be demonstrated if the upper bound of the one-sided 95% confidence interval for the absolute risk difference between groups remains below 5%. Based on these assumptions, an expected event rate of 15% in the control group and 10% in the intervention group, with a one-sided alpha of 0.05 (Zα = 1.645) and a power of 90% (Zβ = 1.282), the required total sample size is 374 patients, with 187 per group.

**Figure 2:**
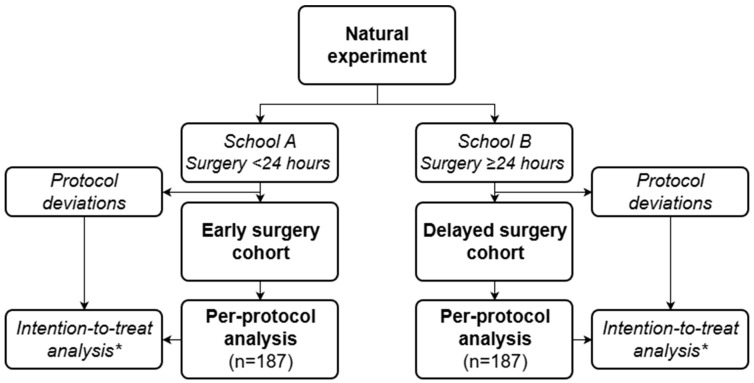
Flowchart of cohort allocation and analysis plan. Patients are quasi-randomly assigned to cohorts based on hospital protocol. Per-protocol analysis includes only patients treated according to their hospital’s policy. * An intention-to-treat analysis will be performed to assess robustness and preserve quasi-randomization, meaning that patients are analyzed according to their initially assigned cohort based on hospital protocol, irrespective of protocol deviations.

As the sample size is based on the per-protocol population, patient inclusion will continue until 187 patients per treatment group have been included who adhered to the protocol of their respective school of treatment (Figure 2). Inclusion in a given school will stop once the required number of per-protocol patients (*n* = 187) is reached in that group. The other school will continue including patients until it also reaches the target of 187 per-protocol patients. As a result, the intention-to-treat population is expected to exceed 374 patients. Since the primary outcome is assessed during hospitalization, no loss to follow-up is anticipated. This approach provides sufficient power to robustly assess non-inferiority within the specified parameters.

### 5.8. Statistical Analysis

#### 5.8.1. Primary Outcome

The primary outcome is postoperative blood transfusion in hip fracture patients on DOACs. To assess non-inferiority of early surgery within 24 h compared to delayed surgery 24 h or more after last DOAC intake, the risk difference in transfusion requirement between both groups will be calculated with a corresponding one-sided 95% CI. Non-inferiority will be concluded if the upper bound of the one-sided 95% CI does not exceed the predefined non-inferiority margin of 5%.

In addition, a multivariable logistic regression analysis will be performed to estimate the adjusted odds ratio (aOR) and associated 95% CI for transfusion requirement, adjusting for potential confounders. Based on literature and expert-opinion, the following variables will be considered for inclusion in the regression model: sex, preoperative Hb, Clinical Frailty Scale, and type of surgical procedure [59,60,61,62]. Given a total sample size of 374 and an expected event rate of 15%, approximately 56 events are anticipated, allowing for inclusion of up to five independent variables, in accordance with standard rules to prevent model overfitting.

The primary analysis will follow a per-protocol approach, in which patients will be analyzed based on adherence to the surgical timing protocol of their respective hospital (i.e., within 24 h for School “A” centers and after 24 h or more for School “B” centers). Patients whose surgical timing deviates from this hospital protocol will be identified and excluded from the per-protocol analysis (Figure 2). In addition, an intention-to-treat (ITT) analysis will be conducted, in which patients will be analyzed according to the timing policy of their admitting hospital, regardless of actual timing. This ITT analysis will serve to assess the robustness of the findings, while preserving the effect of quasi-randomization and limiting the risk of confounding by indication. As a sensitivity analysis, propensity score matching will be applied to further assess robustness of the primary findings. This analysis will allow comparison of results obtained using propensity score matching with those derived from the natural experiment design and provide additional insight into the methodological value of the natural experiment approach.

Lastly, although we do not expect substantial inter-hospital variability, since all participating centers are Dutch level 2 trauma centers with similar hip fracture case volumes and resources, we will assess clustering by center using an intraclass correlation coefficient. If clustering of outcomes is detected (i.e., if some hospitals show greater similarity in outcomes than others), we will perform a multilevel logistic regression analysis to account for center-level effects. If no relevant clustering is found, the standard multivariable logistic regression will be deemed sufficient.

#### 5.8.2. Secondary Outcomes

Secondary outcomes will be analyzed according to the type and distribution of each variable. Continuous variables (i.e., Hb decrease, bleeding index, surgery duration, time to DOAC restart, hospital length of stay) will first be assessed for normality using graphical methods (e.g., histograms). Normally distributed outcomes will be analyzed using parametric tests, specifically independent-sample t-tests. If the distribution is non-normal, non-parametric methods, such as the Mann–Whitney U test, will be applied. Ordinal or categorical variables with more than two categories, such as the number of packed red cell units transfused, will be analyzed using the Chi-square test. Binary outcomes, including thromboembolic events, postoperative complications, and a Hb decrease of more than 2 mmol/L, will be analyzed using Chi-square tests, or Fisher’s exact test in cases where expected cell counts are less than five.

### 5.9. Missing Data

Because of the prospective registration of predefined variables and regular quality checks, we expect minimal missing data. If missing data occur, we will assess their extent and mechanism (missing completely at random, missing at random, or missing not at random). When appropriate, we will apply multiple imputation using chained equations to minimize bias and preserve statistical power.

## 6. Ethics and Dissemination

### 6.1. Ethics

Ethical approval for this study was obtained from the Medical Ethics Committee United (MEC-U), which reviewed the study protocol, ethical considerations, and observational design. The MEC-U concluded that the study does not fall under the scope of the Dutch Medical Research Involving Human Subjects Act (WMO) and issued a formal non-WMO declaration (registration number W25.034). Following this central approval, local feasibility approval was obtained from all participating hospitals.

As a healthcare evaluation study designed as a natural experiment, no changes are made to standard patient care. Treatment allocation is determined by existing clinical protocols in each hospital, and the research team does not influence medical decision-making. Because this study involves retrospective data extraction of predefined prospectively collected clinical data, individual informed consent is not required. Patients are not subjected to any intervention, and no additional procedures or burdens are introduced; therefore, the study poses no risk to participants. All data are pseudonymized prior to analysis to ensure confidentiality and protect patient privacy.

This study is being conducted in accordance with the Declaration of Helsinki and follows the REporting of studies Conducted using Observational Routinely Collected Data (RECORD) guidelines, an extension of the Strengthening the Reporting of Observational Studies in Epidemiology (STROBE) guidelines [63,64].

### 6.2. Dissemination

The results of this study will be disseminated through publication in a peer-reviewed scientific journal, focusing on the primary outcome of postoperative blood transfusion in hip fracture patients on DOACs. Additional manuscripts may be developed for secondary or subgroup analyses addressing other clinically relevant outcomes, such as bleeding complications, thrombotic events, and functional recovery. These will also be submitted to peer-reviewed journals. Presentations at national and international conferences related to trauma surgery, geriatrics, and thrombosis are planned to ensure broad visibility within the clinical and scientific community.

## Data Availability

The data that will be presented in this study are available on request from the corresponding author due to ethical reasons.

## Abbreviations

The following abbreviations are used in this manuscript:

DOAC	Direct oral anticoagulant
EHR	Electronic Health Record
PRBC	Packed red blood cell
HLOS	Hospital length of stay
ITT	Intention-to-treat
AO/OTA	Arbeitsgemeinschaft für Osteosynthesefragen/Orthopedic Trauma Association
eGFR	Estimated Glomerular Filtration Rate
Hb	Hemoglobin
Ht	Hematocrit
BMI	Body Mass Index
SNAQ	Short Nutritional Assessment Questionnaire
CFS	Clinical Frailty Scale
CCI	Charlson Comorbidity Index
ASA	American Society of Anesthesiologists
ADL	Activities of Daily Living
GDPR	General Data Protection Regulation
WMO	Dutch Medical Research Involving Human Subjects Act (Wet medisch-wetenschappelijk onderzoek met mensen)
RECORD	Reporting of studies conducted using observational routinely collected data
STROBE	Strengthening the reporting of observational studies in epidemiology
MEC-U	Medical Ethics Committee United

## Appendix A

**Table A1:** Short overview of pharmacological properties of direct oral anticoagulants prescribed in The Netherlands.

Direct Oral Anticoagulant	Mechanism of Action	Half-Life	Clearance Route
Apixaban	Factor Xa inhibitor	~12 h	~27% renal ~73% through biliary and hepatic metabolism
Edoxaban	Factor Xa inhibitor	10–14 h	~50% renal ~50% through hepatic metabolism and biliary excretion
Rivaroxaban	Factor Xa inhibitor	11–13 h	~36% renal (unchanged) ~30% hepatic metabolism ~34% through fecal excretion
Dabigatran	Factor IIa inhibitor	12–14 h	~80% renal clearance ~20% through hepatic glucuronidation and biliary excretion
